# Radiologic imaging of true diphallia with imperforate anus: A case report

**DOI:** 10.1016/j.radcr.2024.09.100

**Published:** 2024-09-27

**Authors:** Hendra Boy Situmorang, Anggraini Dwi Sensusiati

**Affiliations:** a Department of Radiology, Faculty of Medicine, Universitas Airlangga, Surabaya, Indonesia; b Department of Radiology, Dr. Soetomo General Academic Hospital, Surabaya, Indonesia; c Department of Radiology, Universitas Airlangga Hospital, Surabaya, Indonesia

**Keywords:** Diphallia, Gland duplication, MRI, Voiding cystography, True diphallia

## Abstract

Diphallia, also known as penile duplication, represents a highly unusual congenital abnormality of sex development, occurring in 1 in every 5 million to 1 in thirty million live births. Wecker, in Bologna, Italy, noted the first instance of diphallia in 1609. Meanwhile, no sufficient report has meticulously described the incidence in Indonesia. Generally, this condition is accompanied by duplication or malformation of other organs, such as the urinary tract, anorectal, or vertebrae, whether uncomplicated or complex; therefore, appropriate imaging is paramount to identify the related anatomical structures in order to provide prompt and pertinent management. A wide variety of imaging modalities can be conducted to elucidate this malformation, from the conventional procedure, namely urethrography ultrasound to assess the vessels, to advanced examination, such as magnetic resonance imaging (MRI), to capture the anatomy around the lesion distinctly. Comprehensive imaging enables the surgeon to evaluate and understand the complexity of the anatomical builds. This case report will illustrate diphallia from conventional and advanced perspectives concomitantly of a boy presenting with a hereditary anomaly of 2 penises, each conveying a functional urethra, 2 anal dimples, with a presacral mass between them.

## Introduction

Congenital defect of penile structure refers to a hereditary abnormality that arises as early as embryological development. The presentation can be either aphallia (one of 5,500,000 live births) which is a result of genital tubercle formation failure entailing penile absence, diphallia with an even more infrequent incidence (one in 30,000,000 live births) representing a penile duplication emerging from an incomplete fusion of genital tubercle, or the most exceptional disorder of triphallia with triple phallus formation [1].

Penile duplication, also recognized as diphallia, is a rare sexual development aberration with a broad case diversification – the duplication may occur solely in the penile structure or with neighboring organ involvement, such as the digestive tract and vertebrae. In the case of urinary system complicity, the anatomical anomalies are investigated from the urethra, soft tissue, to the testis. Nonetheless, there has been an account of a female case with diphallia presenting with a double clitoris [2].

An extensively used classification was introduced by Schneider, dividing diphallia into 3 groups, consisting of complete diphallia, bifid diphallia, and duplication of the penile gland. Thereafter, Vilanova designated the fourth classification named pseudodiphallia, referring to a condition of penile duplication with 1 rudimentary and atrophic penis and 1 normal penis [3,4].

A more complex categorization was invented by Kendrick and Kimble, grading diphallia according to the features of penile soft tissue, urethra, and bladder. In contrast, Lisieux classified the malformation based on the embryology, anatomy, clinical manifestation, and surgical aspect into 4 categories – true diphallia, hemi diphallia, pseudo diphallia, and partial duplication [5,6].

A radiology examination can be requested from the fourth week of gestation to explore the urinary and digestive tract abnormality. Although a comprehensive embryologic explanation has yet to be established, it is believed to happen from a novel genital tubercle formation as a structure due to genomic malfunction, trauma, or chemical compound exposure provoking cauda cell mass disturbance in fetal mesoderm. In addition, another report stipulated an involvement of chromosome 46, XY, t (1:14) (p36,3:q24,3) translocation [7,8].

A literature review signified that the majority of cases were accompanied by abnormality of the urogenital system or gastrointestinal tract, with the highest incidence being anal imperforate, which can also be in chaperone with duplication of the terminal ileum, appendix, caecum, colon, or rectum, either with fistulation or not [5].

## Case report

A 4-month-old baby boy was presented to our center with the altered genital structure of 2 penises, a scrotum containing 2 testes, 2 anal dimples with a soft tissue mass between them, and an anal imperforate. He was born assisted by a midwife, with spontaneous cry and normal body weight and length. A history of colostomy diversion procedure was recorded at 2 days old at a referred hospital, with a properly functioning stoma and no complaints of the operation outcome. There was no account of infection, chemical exposure, or trauma towards the mother during gestation, and she had had a routine follow-up with her obstetrician. However, she had been warned about the possibility of the baby having 2 penises upon birth.

**Fig. 1:**
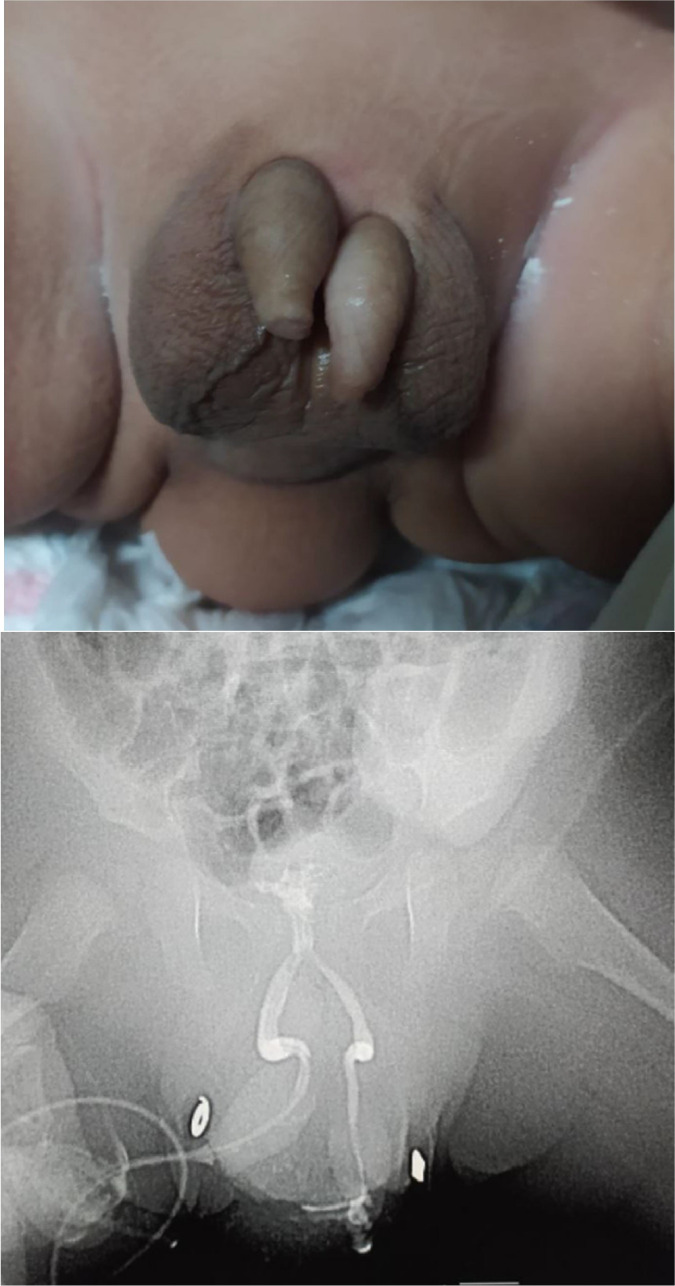
(A) External genitalia of the patient, showing 2 separated penises with a relatively smaller penis on the left side, normal scrotum, 2 anal dimples and a soft tissue mass in between. (B) Voiding cystography elucidates the diameter of both urethrae, with the narrower left urethra relative to the right side converging to the proximal, forming an inverted Y.

**Fig. 2:**
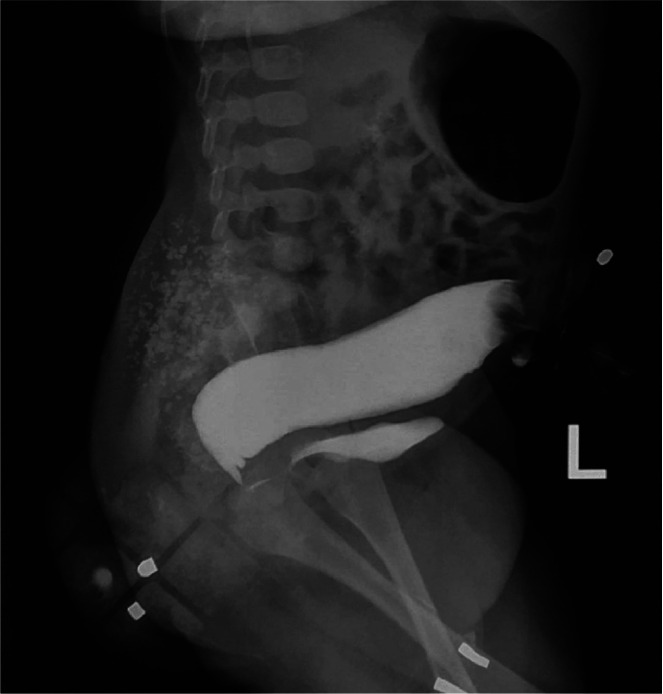
The distal loopography shows the rectovesical fistula with a distal rectal pouch beneath the pubococcygeal line.

The patient could urinate normally using both penises; nevertheless, the second head had reduced urine flow, with early flow cessation and a delay in the urinary outflow. He had been brought for a scheduled visit to the referred hospital for the requested distal loopography, and high-lying anorectal malformation with a rectovesical fistula was discovered on the examination. The patient was then referred to a higher center for a voiding cystography procedure, which revealed diphallia with 2 anterior urethrae, a single posterior urethra, and 1 bladder (Figs. 1 and 2).

**Fig. 3:**
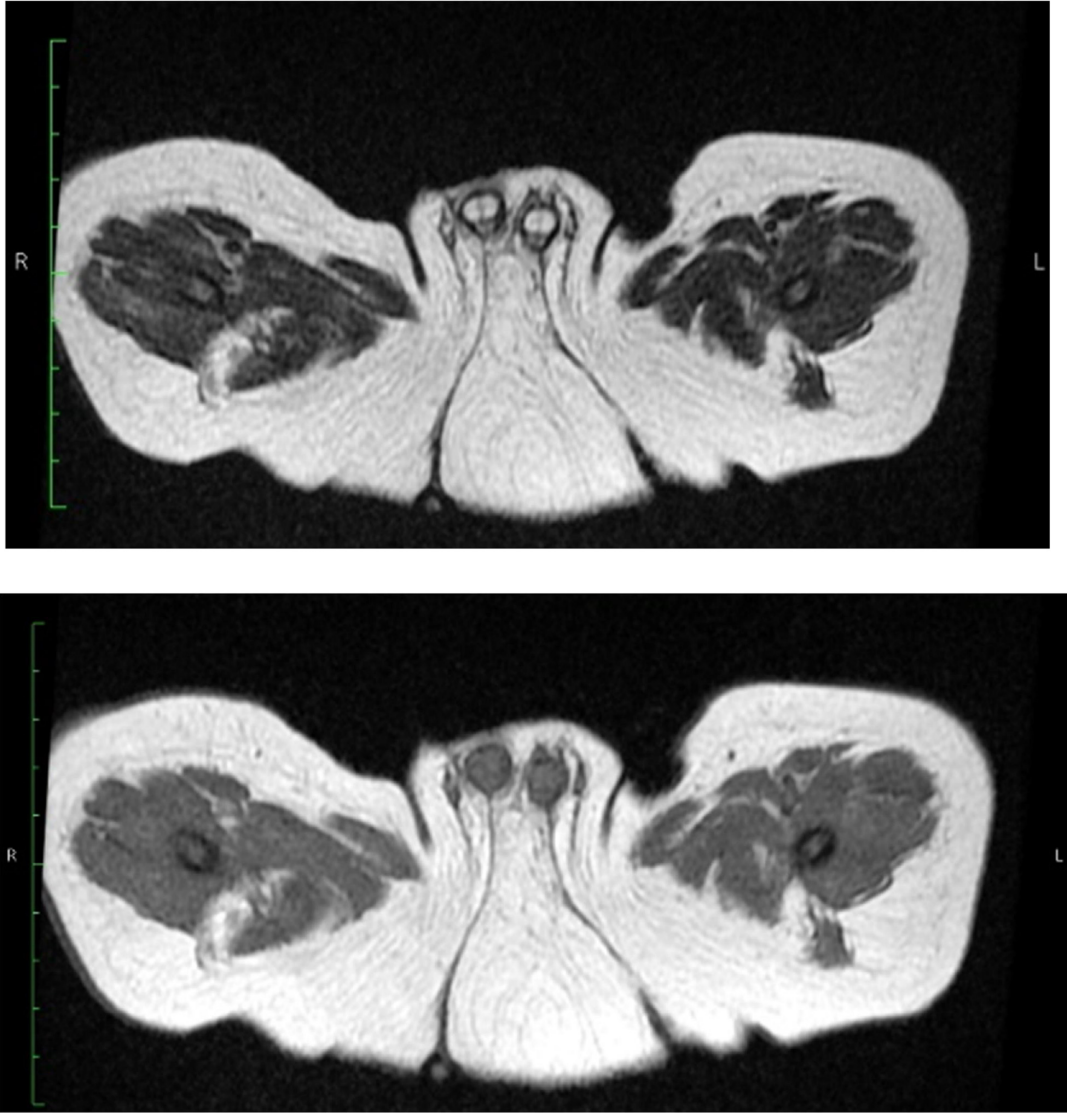
Axial T1-weighted and T2-weighted MR image show the penises and urethrae structures clearly; the presacral mass is seen at the posterior side between the anal dimples.

**Fig. 4:**
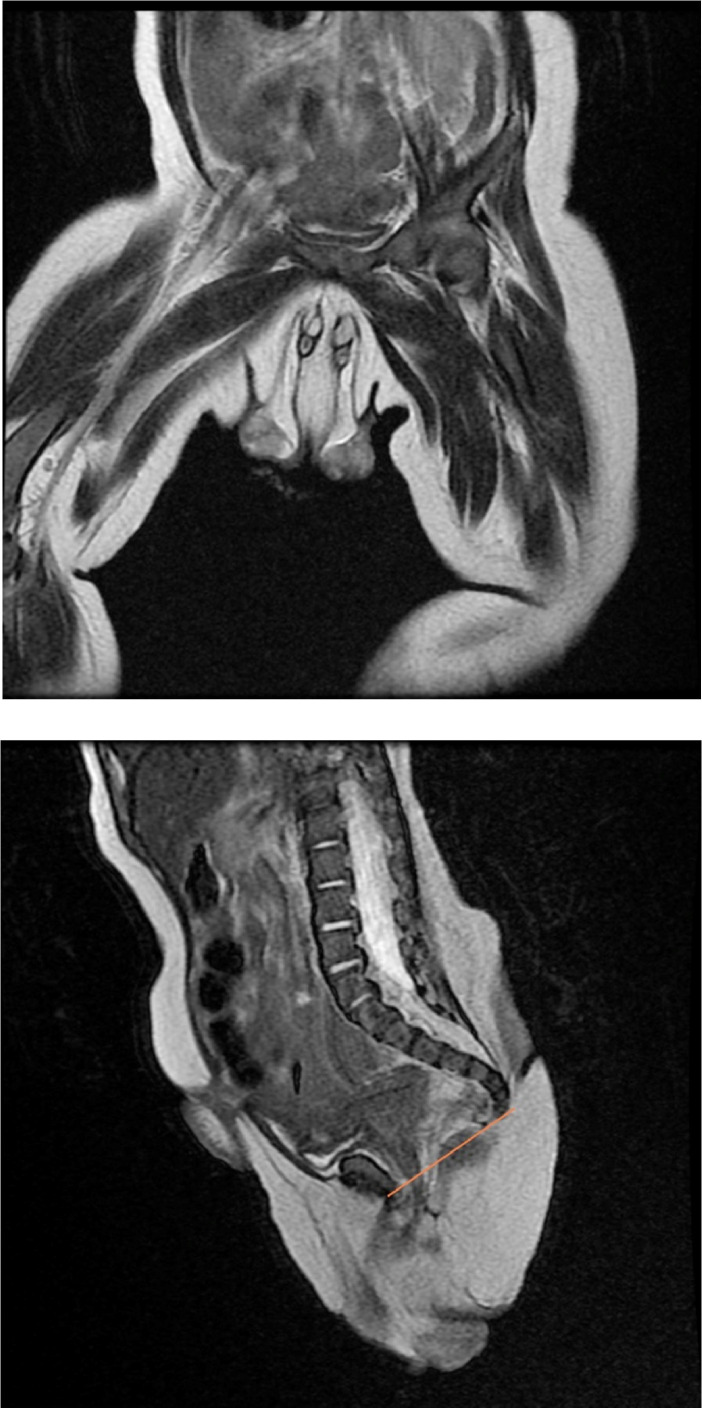
Coronal and sagittal T2-weighted MR image show 2 testes with a well-visualized structure of each penis and the position of the distal rectal pouch compared to the puborectalis muscle.

**Fig. 5:**
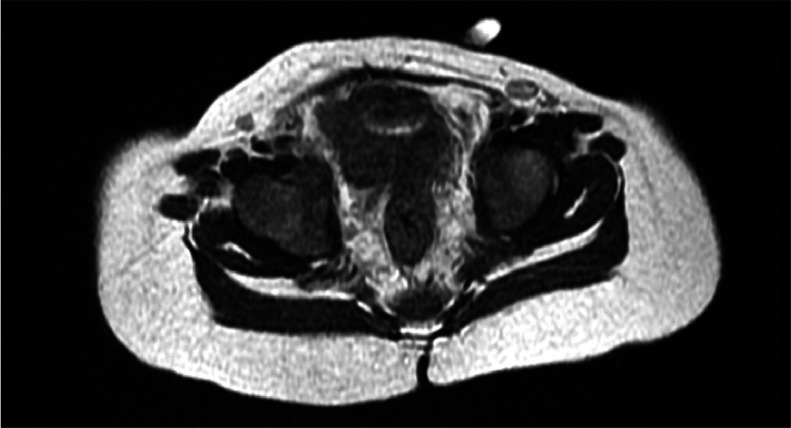
The axial plane T2-weighted MR image shows the complete formation of the sphincter ani in the middle.

The magnetic resonance imaging (MRI) examination was conducted afterwards, picturing 2 cavernous bodies and 1 spongious stem in each penis, 2 anterior urethrae, 2 testes shrouded by the scrotum, a bladder, an anal sphincter in the middle with a presacral mass underneath, and a distal rectal pouch beneath the puborectalis muscle (Fig. 3, Fig. 4, Fig. 5)

## Discussion

The diphallia case in our study was accompanied by anal imperforate, in line with the prior literature stipulating that anorectal malformation was the most prevalent abnormality to follow diphallia. It is mandatory to investigate whether or not the fistula exists and the type of the fistula to decide the most suitable operative procedure for anoplasty surgery. In males, the rectovesical fistula was the most uncommon entity, with an incidence of approximately 15% of all anal atresia [5,6,9].

There has been evidence postulating that in most cases of diphallia, 1 penis would be functioning more improperly than the other due to the chance of structural discrepancy of each penis. Our case confirms this theory, given that the right penis in our patient had better function compared to the other one. Moreover, voiding cystography provided more satisfying clinical information than MRI examination, with a shorter procedure duration and affordable cost [5,8].

During the examination, it is also necessary to consider involving the pediatric surgeon in order to facilitate the commencement of the procedure, particularly in the instrumentation of a younger child. The voiding cystography in our study yielded a picture of a narrower diameter of the left anterior urethra compared to the right side, and the meatus was located in an appropriate position.

Unfortunately, we were unable to evaluate the presence of the rectovesical fistula because we could not fill the bladder. The fistulation was detected during the distal loopography, which is done using an augmented pressure colostogram, an accepted standard to establish the existence of the fistula of a patient with anorectal malformation. Furthermore, another way to investigate the fistula is the ultrasound with a transperineal approach [10,11].

MRI was the advanced technique we used in this case, proposing the evaluation of the more compound structure or the perineal anomaly. In our case study, the definitive diagnosis was concluded through MRI examination due to the presence of the complete 3 corpora in each penis, encompassing 2 cavernous bodies and a corpus spongiosum with a urethra between the structures [12].

According to the classification by Kendrick and Kimble, the most acceptable category for our patient is class 1E⍺ given the appearance of 3 corpora in each penis. Meanwhile, based on the Lisieux categorization, true diphallia is preferred due to the sagittal duplication with ventrally located normal urethrae, with both urethrae functioning correctly, although 1 penis was superior to the other. Individualistic management was obligatory in this case [5,6].

In the T2 sequence, the penile body was explicitly identified as a shaft containing 2 corpora cavernosa with 1 corpus spongiosum and a urethra that lies between the 3 structures, mimicking a target-shaped appearance. Alongside the mentioned anatomy, there was also a presacral soft tissue mass measuring around 1.7 × 0.7 × 3.5 cm between the anal dimples, which appeared isointense in both T1-weighted imaging (T1WI) and T2WI sequences with homogenous contrast enhancement upon contrast administration.

On the coronal plane of the T2 sequence, the true diphallia was evident, communicating with 2 testes inside the scrotum, whereas, on the sagittal plane, the distal rectal pouch was seen below the puborectalis muscle, giving the impression of low-level anorectal malformation.

Interestingly, each case of diphallia has always had its uniqueness; therefore, the chosen imaging modality must have an outstanding ability to represent the adjacent structures to foster accurate surgical management to take place. In our case, not every abnormality could be explained through MRI, yet either contrast administration or fat sat sequence was unable to yield additional information.

The conventional technique, namely voiding cystography, was an excellent examination in raising the conclusion for the complaint of the patient's guardian and the patient's Y-shaped urethra. Nevertheless, MRI was able to grant a more precise diagnosis with a thorough description of the surrounding anatomy; hence, we could settle on the definitive diagnosis. Conscientious information was expected to aid in avoiding all redundant procedures and shortening the hospitalization duration. Our patient was scheduled to undergo a left-sided penis excision followed by corporal body penis reconstruction.

## Conclusion

Our case report has detailed the value of radiologic examination in the case of diphallia, which is a broad-spectrum diagnosis that requires a comprehensive knowledge of imaging to elucidate the most appropriate modality. In this case, the conventional study was necessary to depict the structure of the urethra; in general, MRI examination played a crucial role in assessing the extensive topography of diphallia cases.

## Ethical clearance statement

This paper has also obtained mandatory ethical clearance from Ethical Commitee of our hospital.

## Patient consent

The patient has consented to this paper's publication on the condition of requisite anonymity of obtained data.
